# Martensitic Transformation Temperatures and Hall Effect in Ni_47−x_Mn_41+x_In_12_ (x = 0, 1, 2) Alloys

**DOI:** 10.3390/ma16020672

**Published:** 2023-01-10

**Authors:** Vyacheslav V. Marchenkov, Sabina M. Emelyanova, Elena B. Marchenkova

**Affiliations:** M.N. Mikheev Institute of Metal Physics of the Ural Branch of the Russian Academy of Sciences, 620108 Ekaterinburg, Russia

**Keywords:** magnetocaloric effect, Heusler alloys Ni-Mn-In, Hall effect, valence electron concentration *e*/*a*, concentration of charge carriers *n*

## Abstract

At present, the question of the relationship between the characteristic martensitic transformation temperatures (MTT) and the electronic parameters of a system has not been fully studied. In the present work, an attempt to establish a similar relationship using the example of the concentration of charge carriers, *n*, was made. The field dependences of Hall resistivity *ρ_H_* and magnetization *M* of the magnetocaloric Ni_47−x_Mn_41+x_In_12_ (x = 0, 1, 2) alloys were measured at *T* = 4.2 K and in magnetic fields of up to 80 kOe. The MTT were obtained from the temperature dependences of electrical resistivity and magnetization. It was observed that the MTT correlate strongly with both the valence electron concentration *e*/*a* and the electronic transport characteristics, which are the coefficient of the normal (NHE) *R*_0_ and anomalous (AHE) *R_S_* Hall effect and the concentration of charge carriers *n*.

## 1. Introduction

Despite the fact that Heusler alloys were discovered by F. Heusler in 1903, the synthesis and study of their physical properties are still of great interest [1]. This is largely due to the fact that many unusual phenomena, states, and useful functional properties can be observed in Heusler alloys: half-metallic ferromagnetism [2], the state of a spin gapless semiconductor [3], topological semimetals [4,5,6], large thermoelectric effect, and unusual thermal properties [7,8,9], shape memory effect [10,11,12], magnetocaloric effect (MCE) [13,14,15,16], etc. Most of these phenomena arise due to the peculiarities of their crystal and electronic structure and the magnetic state.

If the role of the parameters of the crystal structure, electronic and magnetic characteristics in the formation of the states of a half-metallic ferromagnet, a spin gapless semiconductor, and a topological semimetal has been studied in sufficient detail [5], then the relationship between the parameters of the electronic structure and shape memory effects and MCE has not been studied enough, since such information is practically absent in the modern scientific literature.

Heusler alloys with a large MCE, which, as a rule, is observed near the martensitic transformation temperatures (MTT) [17], are of particular practical interest. Despite the huge number of publications in this area, there are still plenty of unresolved problems. For example, it is known that there is a certain correlation between the MTT and the parameter *e*/*a*, where *e* is the number of valence electrons, and *a* is the number of atoms in the unit cell. As a rule, the values of MTT increase with an increase in the parameter *e*/*a* [18,19]. However, there are some cases when MTT decreases with an increase in *e*/*a* [20,21] or such dependences are not monotonous [22,23]. This is largely because the size of the unit cell can be different at the same value of *e*/*a*. This leads to the situation when, even at the same *e*/*a*, the electron density *N*, i.e., the number of valence electrons per cell, can be different [24].

For the first time, this circumstance was noticed in [24], where nonmonotonic dependences of MTT on *e*/*a* and the volume of the unit cell *V_cell_* were observed, but “good” monotonic changes of MTT on the electron density *N* were observed. Note that in [24], the parameters of the crystal lattice, and then *V_cell_*, were determined from X-ray data at room temperature; however, as a rule, alloys are in a two-phase state, i.e., they contain both austenitic and martensitic phases [25]. In addition, the structure and physical properties of compounds of the same composition can vary greatly depending on the method of preparation [26,27,28].

Thus, the question arises of finding the electronic parameters of a system that characterize this specific compound and that can be measured experimentally for it. Apparently, such a parameter should also correlate with the MTT. In our opinion, the concentration of current carriers *n* could be such a parameter.

Heusler alloys based on Ni-Mn-In were selected as the objects of this study, because martensitic transformations are observed in them near room temperature (see, for example, [14]), i.e., the austenitic and martensitic phases coexist. Heusler alloys based on the Ni-Mn-In system have a unique feature: they can simultaneously realize both direct and reverse MCE at room temperature [29]. Moreover, they are very sensitive to element substitution and stoichiometric variations. For example, in [30], for the Ni_50_Mn_35_In_15_ alloy, a giant value of the isothermal entropy change ΔS = 35.8 J/(kg·K) (H = 50 kOe) was found, but in [31], it was shown that in the alloys based on Ni_50_Mn_35_In_15−x_Si_x_ (1 ≤ x ≤ 5), the largest ΔS value (this is the largest value of the inverse MCE of all the alloys ever studied) was found in the Ni_50_Mn_35_In_12_Si_3_ alloy, for which ΔS = 124 J/(kg·K) at the temperature T = 239 K (H = 50 kOe). The Hall effect in Ni-Mn-In-based alloys was studied for the first time in 2009 [32]. In [33], the main type of charge carrier was determined (electrons) for the Ni-Mn-In-Si alloys. Despite the fact that the Hall effect in Ni-Mn-In-based alloys [33,34,35] has been investigated in the modern literature, there is practically no data on the relationship between MTT and electronic characteristics, in particular, the concentration of current carriers *n*, with the exception of the report [36]. The Heusler alloys with compositions Ni_47−x_Mn_41+x_In_12_ (x = 0, 1, 2) were particularly chosen, since the magnetostructural phase transition as well as MCE were observed in these alloys [37].

Thus, the aim of this work is to study the Hall effect and evaluate the concentration of charge carriers *n*, looking for the relationship between the MTT and parameter *n* in the Ni_47−x_Mn_41+x_In_12_ (x = 0, 1, 2) Heusler alloys.

## 2. Materials and Methods

**Figure 1:**
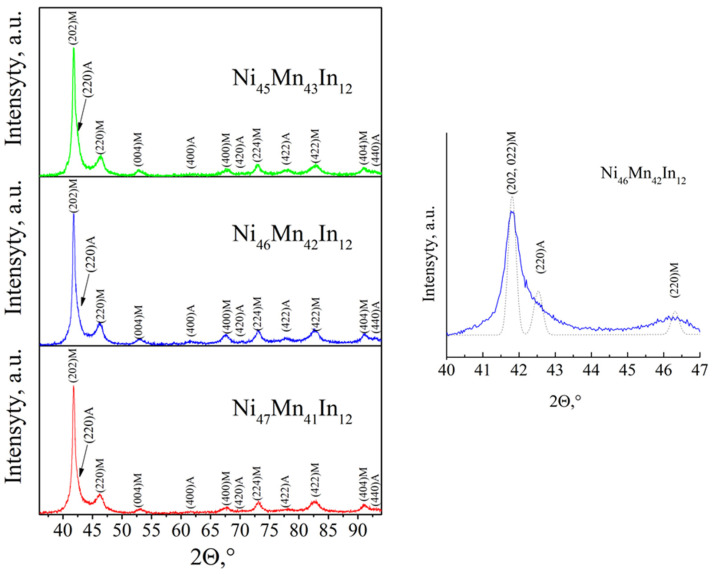
XRD patterns for Ni_45_Mn_43_In_12_, Ni_46_Mn_42_In_12_, and Ni_47_Mn_41_In_12_ alloys at room temperature. Here, A and M are austenite and martensite, respectively. On the right panel, the XRD pattern of Ni_46_Mn_42_In_12_ alloy is represented. The splitting of (220) austenite reflection onto the (220) and (202, 022) martensite reflections is shown by the dotted line.

The polycrystalline alloys were melted in an arc furnace in an inert argon atmosphere. Then, the alloys were annealed according to the following regime: exposure at 1100 K for 24 h, followed by cooling with a furnace. The samples for research were cut from the obtained ingots using electric spark cutting. The elemental analysis was carried out on a Quanta-200 Pegasus (FEI Company, Eindhoven, the Netherlands) scanning electron microscope equipped with EDAX (Energy Dispersive X-ray Analyser) spectrometer and EBSD (Electron Backscattered Diffraction) system. XRD analysis was performed at room temperature at the Center of Collective Use, M.N. Mikheev Institute of Metal Physics, UB RAS. X-ray diffraction phase analysis was carried out at room temperature; the range of angles varied from 30° to 100° (Figure 1).

As a result of X-ray diffraction studies, it was found that all alloys are in a two-phase state, i.e., the austenite and martensite phases are simultaneously present in the structure of the alloys (Table 1).

The magnetization was measured in magnetic fields of up to 70 kOe at 4.2 K and in the temperature range from 50 to 330 K. The electrical resistivity and the Hall effect were measured using the standard four-probe method at direct current in the temperature range from 75 to 375 K and at 4.2 K in magnetic fields of up to 80 kOe, respectively. All samples for measuring the Hall effect were in the form of plates with dimensions 0.5 mm × 1.5 mm × 4.5 mm; thus, the ratio of dimensions 1:3:9 is fulfilled (demagnetization factor *F* = 1).

## 3. Results and Discussion

**Figure 2:**
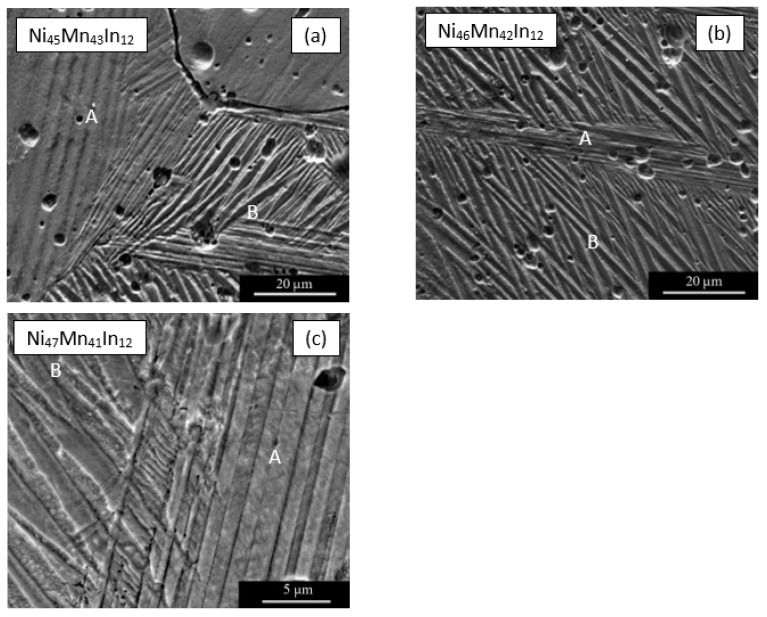
Electron microscopy images of the martensite structures in the alloys at room temperature: (**a**) Ni_45_Mn_43_In_12_, (**b**) Ni_46_Mn_42_In_12_, (**c**) Ni_47_Mn_41_In_12_. The letters A and B denote different morphological types of martensitic crystals.

Using electron microscopy, it was established (Figure 2) that in all alloys, martensite has a predominant morphology in the form of a packet-pyramidal hierarchy of finely twinned coherent crystals. Two morphological types of martensite crystals are distinguished: pairwise twinned lamellar martensite within the package (A) and truss-shaped pairwise twinned martensite with wedge-shaped conjugations of adjacent crystals (B). The formation of a hierarchy of twinned martensite crystals is due to their coherent adaptation and the minimization of elastic energy during thermoelastic martensitic transformation.

Elemental analysis of the composition of the investigated alloys was carried out along the edges and in the center of the sample; in each case, three measurements were made (Table 2). The examination of the elemental analysis showed that the real composition of all alloys corresponded to the specified with good accuracy.

**Figure 3:**
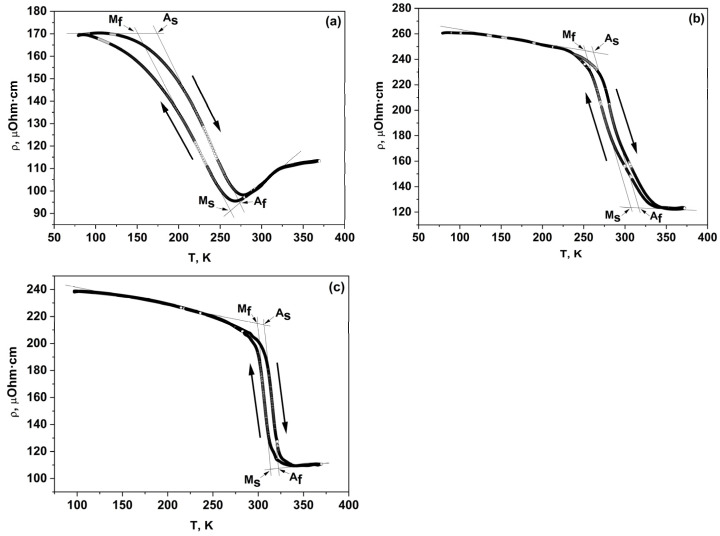
Temperature dependences of electrical resistivity ρ(T): (**a**) Ni_45_Mn_43_In_12_, (**b**) Ni_46_Mn_42_In_12_, (**c**) Ni_47_Mn_41_In_12_.

Figure 3 shows the temperature dependences of electroresistivity ρ(T). It can be seen that at low temperatures, the resistivity value for all alloys is ~(170–260) μΩ cm. With increasing temperature, the resistivity drops sharply, decreasing to values of ~(95–120) μΩ cm. In this case, temperature hysteresis is observed in ρ(T) dependences, which may indicate the implementation of a first-order phase transition in the alloys studied [37]. This behavior of resistivity can be explained by the fact that at low temperatures, there is a large amount of martensite. Electron scattering on martensite leads to a large contribution to resistivity [38,39,40]. With temperature, the amount of the martensite phase decreases, and consequently, the resistivity decreases [38,39,40].

**Figure 4:**
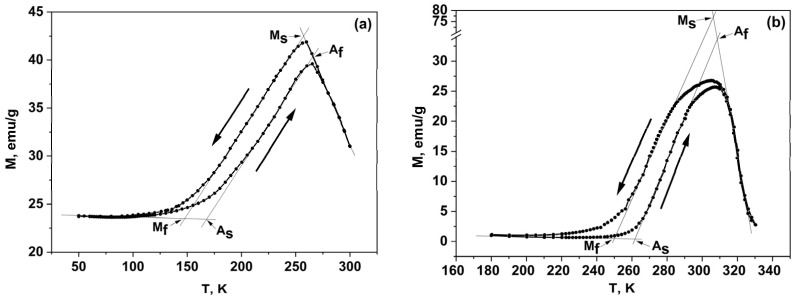

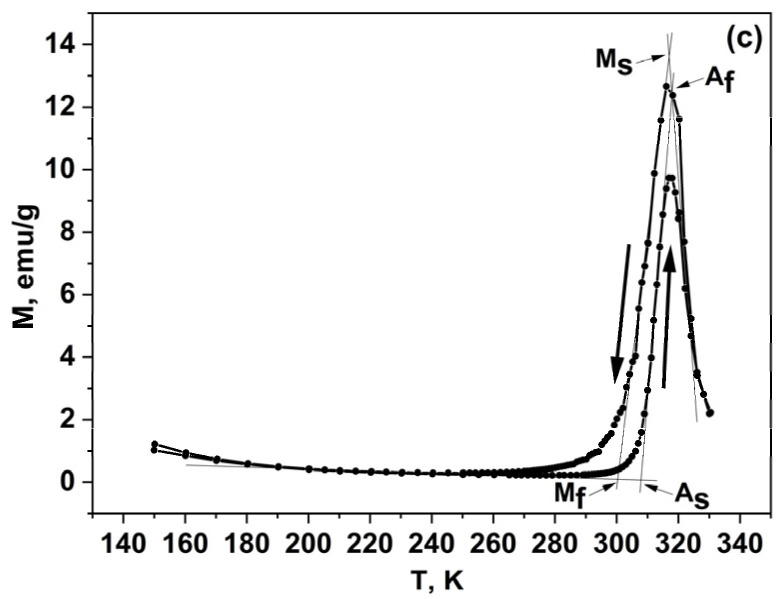
Temperature dependences of magnetization M(T) in the magnetic field H = 1 kOe: (**a**) Ni_45_Mn_43_In_12_, (**b**) Ni_46_Mn_42_In_12_, (**c**) Ni_47_Mn_41_In_12_.

Figure 4 shows the temperature dependences of magnetization for the alloys. These dependences were measured in the magnetic field H = 1 kOe upon cooling and heating (in the figure shown by arrows). It is obvious that for all investigated alloys, the form of these dependences is typical. Obviously, the M(T) curves exhibit minima and maxima, along with the hysteresis in the region of phase transformation temperatures (*A_S_*, *A_F_*, *M_S_*, *M_F_*).

On heating, the transition of paramagnetic martensite to ferromagnetic austenite occurs, which is accompanied by a jump in magnetization.

The MTT were determined from the M(T) and ρ(T) dependences using the method of tangents [41]; the values obtained are shown in Table 3. The calculation of the valence electron concentration *e*/*a* for the unit cell was carried out according to Equation (1) [42]:

(1) $$e/a=(C_{A}\times Z_{A})+(C_{B}\times Z_{B})+(C_{D}\times Z_{D}),$$

where *C_A_*, *C_B_*, and *C_D_* are concentrations of elements *A*, *B*, and *D*, and *Z_A_*, *Z_B_*, and *Z_D_* are the number of external (valence) electrons for the elements *A*, *B*, and *D*. For nickel, manganese, and indium atoms, the number of valence electrons is 10 (3d^8^4s^2^), 7 (3d^6^4s^1^), and 3 (5s^2^5p^1^), respectively. The values of *e*/*a* are 7.87, 7.9, and 7.93 for Ni_45_Mn_43_In_12_, Ni_46_Mn_42_In_12_, and Ni_47_Mn_41_In_12_ alloys, respectively.

It is obvious that with a decrease in the nickel content, a decrease in the valence electron concentration *e*/*a* occurs, and in addition, a shift of the martensitic transformation to the region of low temperatures is observed. Similar behavior was observed in the Ni_2+x_Mn_1−x_Ga [43,44] and Ni_0.5_Mn_0.5−x_Sn_x_ (x = 0.05; 0.10; 0.13; 0.15) alloys [45]. A decrease in MTT leads to an expansion of the existence region of the austenite phase since the austenite magnetization is greater than the martensite one. The same situation is observed for alloys of the Ni-Mn-Sb system, in contrast to the Ni-Mn-Ga system, in which the saturation magnetization of the martensitic phase is higher than that of the austenitic phase [46].

**Figure 5:**
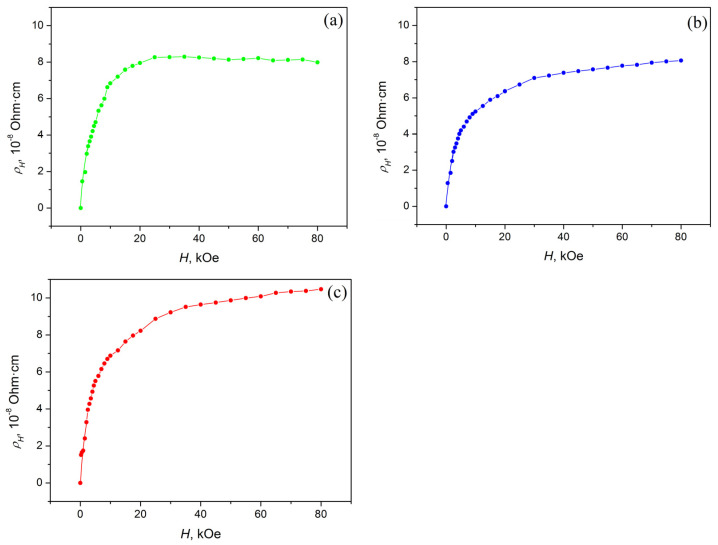
Field dependences of the Hall resistivity *ρ_H_*(*H*) at *T* = 4.2 K: (**a**) Ni_45_Mn_43_In_12_, (**b**) Ni_46_Mn_42_In_12_, (**c**) Ni_47_Mn_41_In_12_.

**Figure 6:**
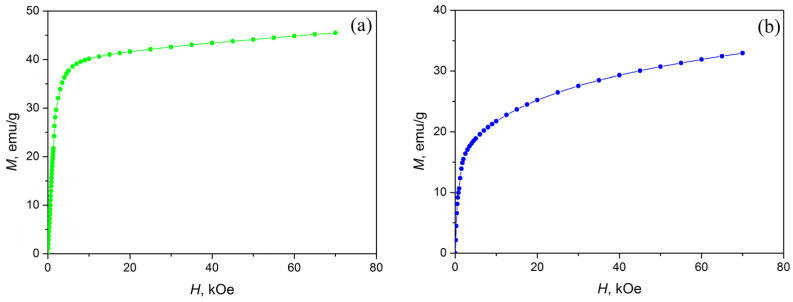

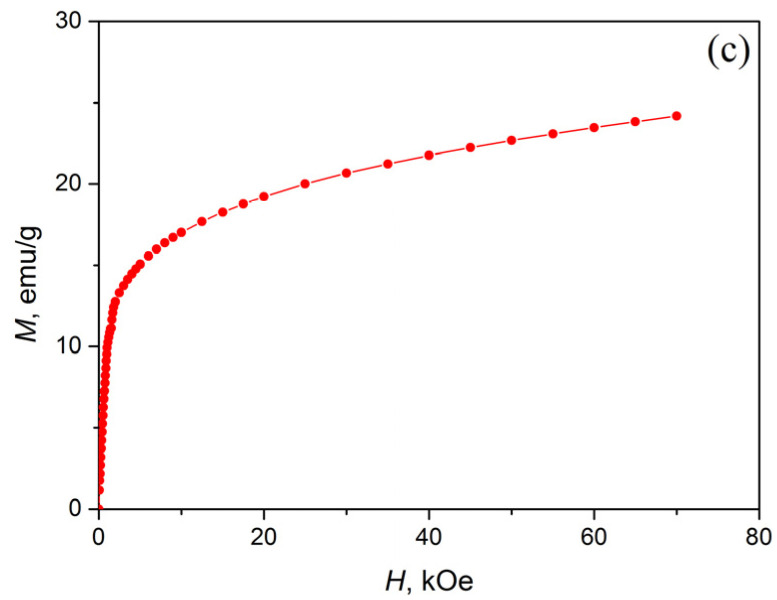
Field dependences of magnetization *M*(*H*) at *T* = 4.2 K: (**a**) Ni_45_Mn_43_In_12_, (**b**) Ni_46_Mn_42_In_12_, (**c**) Ni_47_Mn_41_In_12_.

Due to the fact that the investigated alloys Ni_47−x_Mn_41+x_In_12_ (x = 0; 1; 2) are in a ferromagnetic state at low temperatures, at T = 4.2 K, both normal and anomalous Hall effects will be observed in them. The separation of the normal and anomalous components of the Hall effect was carried out using *ρ_H_*(*H*) (Figure 5) and *M*(*H*) (Figure 6) dependences. It is obvious that the above-mentioned dependences have two well-distinguishable regions of magnetic fields: the region of technical magnetization (H < 20 kOe) and the region of the paraprocess (at higher fields). All investigated alloys at H > 20 kOe approach saturation. In this region of magnetic fields, the process of technical magnetization practically ends, and the alloys pass into a single-domain state.

The coefficients of normal *R*_0_ and anomalous *R_S_* Hall effect were determined using Equation (2) from the field dependences of *ρ_H_*(*H*) and *M*(*H*) in the region of the paraprocess:

(2) $$\frac{\rho_{H}}{H}=R_{0}+\frac{4\pi R_{S}^{*}M}{H},$$

where $R_{S}^{*}=R_{s}+(1-F)\times R_{0},$ and *F* is a demagnetization factor, which equals 1 for the samples studied; hence, $R_{S}^{*}\approx R_{S}$ in our case. The first term in Equation (2) describes the normal Hall effect (NHE), which is caused by the action of the Lorentz force on the charge carriers and is proportional to the applied magnetic field. The second term in Equation (2) is determined by the so-called anomalous Hall effect (AHE), related to the influence of spin-orbit interaction (SOI).

The concentration of current carriers *n* was determined using Equation (3):

(3) $$R_{0}=\frac{1}{nec},$$

where *c* is the light velocity and *e* is the charge of the electron.

**Figure 7:**
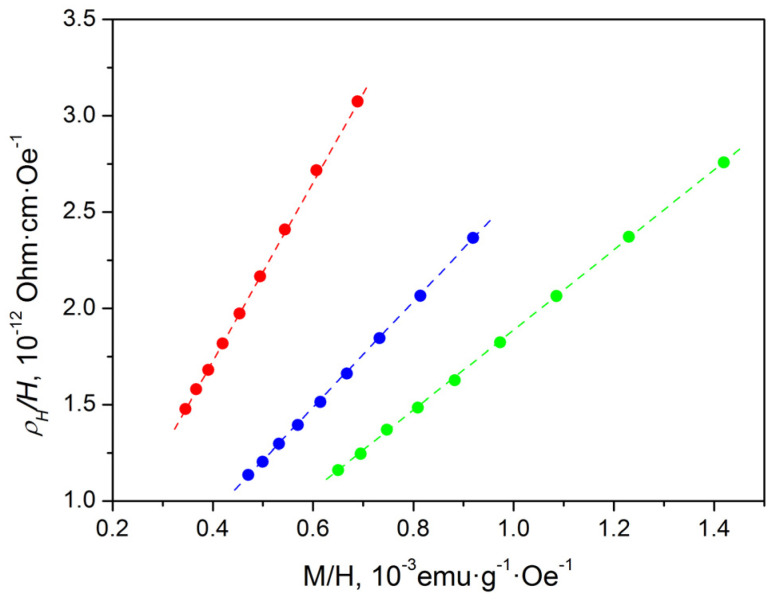
Dependences of *ρ_H_*(*H*) on *M*/*H* for Ni_47−x_Mn_41+x_In_12_ (x = 0; 1; 2) alloys at *T* = 4.2 K: green circles—Ni_45_Mn_43_In_12_, blue circles—Ni_46_Mn_42_In_12_, red circles—Ni_47_Mn_41_In_12_.

Figure 5 and Figure 6 show that Equation (2) is valid for all investigated alloys in high magnetic fields (H > 20 kOe). The NHE *R*_0_ and AHE *R_S_* coefficients were obtained using *ρ_H_*(*H*) (Figure 5) and *M*(*H*) (Figure 6) dependences, and Equation (2). The results obtained are presented in Table 4 and Figure 7.

**Figure 8:**
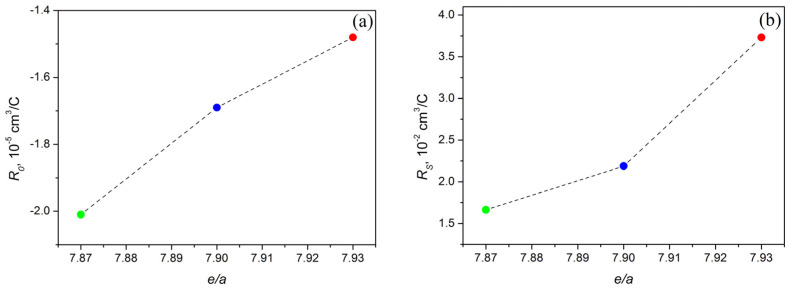
Dependences of the normal *R*_0_ (**a**) and anomalous *R_S_* (**b**) Hall coefficients on the *e*/*a* ratio: green circle—Ni_45_Mn_43_In_12_, blue circle—Ni_46_Mn_42_In_12_, red circle—Ni_47_Mn_41_In_12_.

The dependences of the NHE and AHE coefficients on the valence electron concentration *e*/*a* for the Ni_47−x_Mn_41+x_In_12_ (x = 0; 1; 2) alloys are shown in Figure 8. Obviously, the NHE coefficient is negative; therefore, electrons are the main type of charge carriers. In contrast, the AHE coefficient is positive; its absolute value exceeds the values for NHE by 3 orders of magnitude. Both NHE and AHE decrease with the growth of *e*/*a* ratio. The results obtained are in qualitative agreement with the results for the Ni-Mn-Sb system alloys [36].

Accurate determination of the concentration of charge carriers in Heusler alloys is a rather challenging task, since this requires the data on the Fermi surface topology of the investigated alloy, as well as the data on the mobility of charge carriers belonging to certain sheets of the Fermi surface. This is due to the fact that the Fermi surface of Heusler alloys has a complex topology and contains sheets of both electron and hole types. However, in [47,48], it was shown that even in complex compounds, using a one-band model for estimating the concentration of charge carriers makes it possible to qualitatively track the changes in the electronic characteristics. Taking the above into account, the one-band model [49] was also used in this paper.

**Figure 9:**
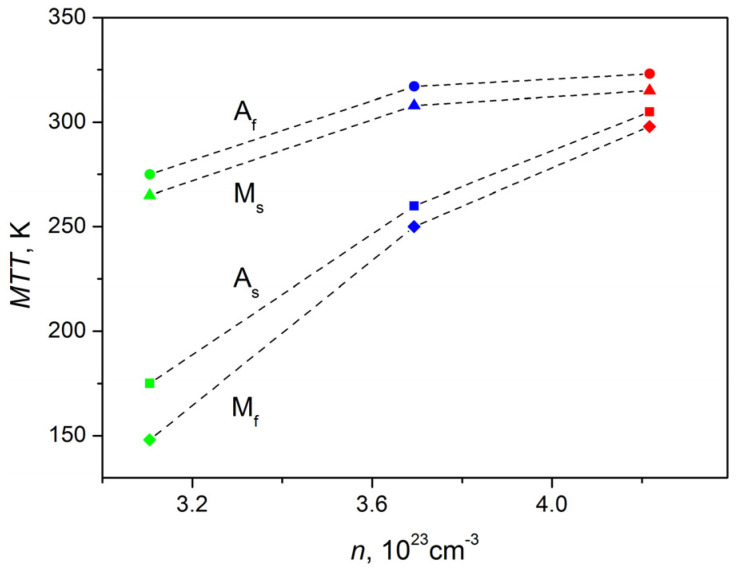
Dependences of the MTT on the concentration of current carriers: green symbols—Ni_45_Mn_43_In_12_, blue symbols—Ni_46_Mn_42_In_12_, red symbols—Ni_47_Mn_41_In_12_; squares—*A_S_*, circles—*A_F_*, triangles—*M_S_*, rhombuses—*M_F_*.

The dependence between MTT and the concentration of charge carriers is shown in Figure 9. It is obvious that the values of MTT decrease monotonically with an increase in the concentration of charge carriers *n*. Considering that a similar dependence was observed for the Ni-Mn-Sb system alloys [36], it can be assumed that such dependence can also be observed in other alloys with a magnetocaloric effect.

It is known that the magnetic shape memory effect (MSME) and the large MCE in Heusler alloys are strongly associated with the magnetostructural transformations and manifest themselves the most near the phase transformation temperatures, in particular, near MTT. The crystal structure and magnetic and electronic subsystems in Heusler compounds are strongly interconnected and dependent on each other. Even quite small changes of about 1–2 at.% in the Ni/Mn ratio in our Ni_47−x_Mn_41+x_In_12_ alloys (x = 0, 1, 2) lead to huge changes in MTT values (Table 3), electrical resistivity (Figure 3), and magnetization values (Figure 4). What is the reason for such drastic changes? One possible explanation could be a change in the density of electronic states (DOS) at the Fermi level *E*_F_, and/or a shift of *E*_F_ with a change in the Ni/Mn ratio. It is obvious that a change in DOS at *E*_F_ will lead to a change in the electronic kinetic coefficients and parameters, particularly in the concentration of current carriers *n*. To verify this assumption, it is desirable to carry out calculations of the electronic band structure for the alloys studied in this paper.

In the present paper, the Hall effect was studied at the temperature T = 4.2 K, while the MTT values were in the region of higher temperatures. To accurately determine the parameters of the electronic subsystem and establish their relationship with MTT, it is necessary to study the Hall effect and other electronic transport properties at temperatures comparable to MTT. However, in the present paper, as in [36], a relationship between MTT and the concentration of charge carriers *n* was established.

It is important to note Ref. [50], where Ni_55−x_Co_x_Fe_18_Ga_27_ (x = 6, 10, 12, 15, 20) single-crystalline fibers with a Heusler structure L2_1_ were studied. The unusual non-hysteretic superelasticity of NiCoFeGa single crystals was observed. It was suggested that the supercritical elasticity originates from a continuous phase transition [50]. It should be noted that in our Ni-Mn-In alloys, the concentration of charge carriers *n* and MTT increase with an increase in *e*/*a*. Taking into account Ref. [50], it is quite interesting to follow the change of *e*/*a* to maximum possible values from the point of existence of first-order martensitic transformation.

## 4. Conclusions

Thus, it was found that the values of the coefficient of the NHE *R*_0_ are negative for all investigated alloys; therefore, electrons are the main charge carriers. In addition, the *R*_0_ values increase with an increase in the nickel content in the alloy, i.e., as the valence electron concentration *e*/*a* increases. The coefficients of the AHE *R*_S_ turned out to be positive, and their values increased with increasing *e*/*a* as well. It was found that as the values of *e*/*a* increase, the concentration of charge carriers *n* as well as the MTT increase. Thus, as a result of this work, for the alloys studied, a relationship between the concentration of charge carriers *n* and MTT was found; that is, MTT increases with the growth of *n*.

## Figures and Tables

**Table 1:** Phase composition of the Ni_47−x_Mn_41+x_In_12_ (x = 0; 1; 2) alloys. The austenite and martensite phases are marked by A and M, respectively.

Alloy	Content of Phases, at.%	
	Cubic (A)	Tetragonal (M)
Ni_45_Mn_43_In_12_	34.1	65.9
Ni_46_Mn_42_In_12_	36.9	63.1
Ni_47_Mn_41_In_12_	39.5	60.5

**Table 2:** Chemical analysis results of the Ni_47−x_Mn_41+x_In_12_ (x = 0; 1; 2) alloys.

Alloy	Content of Each Element, at.%		
	Ni	Mn	In
Ni_45_Mn_43_In_12_	45.21	42.75	12.04
Ni_46_Mn_42_In_12_	46.11	41.71	12.18
Ni_47_Mn_41_In_12_	46.58	41.17	12.25

**Table 3:** Phase transition temperatures for the Ni_47−x_Mn_41+x_In_12_ (x = 0; 1; 2) alloys. The temperatures at the beginning and end of austenitic (*A_s_*, *A_f_*) and martensitic (*M_s_*, *M_f_*) transformation, respectively.

Alloy	*A_s_*, K	*A_f_*, K	*M_s_*, K	*M_f_*, K
According to the temperature dependences of electrical resistivity ρ(T)				
Ni_45_Mn_43_In_12_	175	275	265	148
Ni_46_Mn_42_In_12_	260	317	308	250
Ni_47_Mn_41_In_12_	305	323	315	298
According to the temperature dependences of magnetization M(T)				
Ni_45_Mn_43_In_12_	170	265	258	148
Ni_46_Mn_42_In_12_	263	310	307	252
Ni_47_Mn_41_In_12_	308	318	317	302

**Table 4:** The valence electron concentration *e*/*a*, normal *R*_0_, and anomalous *R_S_* Hall effect coefficients and concentration of charge carriers *n* for the Ni_47−x_Mn_41+x_In_12_ (x = 0; 1; 2) alloys.

Alloy	*e*/*a*	*R*_0_, 10^−5^ cm^3^/C	*R_S_*, 10^−2^ cm^3^/C	*n,* 10^23^ 1/cm^3^
Ni_45_Mn_43_In_12_	7.87	−2.01	1.66	3.11
Ni_46_Mn_42_In_12_	7.9	−1.69	2.19	3.69
Ni_47_Mn_41_In_12_	7.93	−1.48	3.73	4.22

## Data Availability

Not applicable.
